# Fruit-Derived Polyphenol Supplementation for Athlete Recovery and Performance

**DOI:** 10.1007/s40279-018-0998-x

**Published:** 2019-01-22

**Authors:** Joanna Bowtell, Vincent Kelly

**Affiliations:** 1 Sport and Health Sciences, College of Life and Environmental Sciences, University of Exeter, Exeter, EX1 2LU UK; 2 School of Human Movement and Nutrition Sciences, University of Queensland, St Lucia, QLD Australia; 3 School of Exercise and Nutrition Sciences, Queensland University of Technology, Brisbane, QLD Australia

## Abstract

Polyphenols are characterised structurally by two or more hydroxyl groups attached to one or more benzene rings, and provide the taste and colour characteristics of fruits and vegetables. They are radical scavengers and metal chelators, but due to their low concentration in biological fluids in vivo their antioxidant properties seem to be related to enhanced endogenous antioxidant capacity induced via signalling through the Nrf2 pathway. Polyphenols also seem to possess anti-inflammatory properties and have been shown to enhance vascular function via nitric oxide-mediated mechanisms. As a consequence, there is a rationale for supplementation with fruit-derived polyphenols both to enhance exercise performance, since excess reactive oxygen species generation has been implicated in fatigue development, and to enhance recovery from muscle damage induced by intensive exercise due to the involvement of inflammation and oxidative damage within muscle. Current evidence would suggest that acute supplementation with ~ 300 mg polyphenols 1–2 h prior to exercise may enhance exercise capacity and/or performance during endurance and repeated sprint exercise via antioxidant and vascular mechanisms. However, only a small number of studies have been performed to date, some with methodological limitations, and more research is needed to confirm these findings. A larger body of evidence suggests that supplementation with > 1000 mg polyphenols per day for 3 or more days prior to and following exercise will enhance recovery following muscle damage via antioxidant and anti-inflammatory mechanisms. The many remaining unanswered questions within the field of polyphenol research and exercise performance and recovery are highlighted within this review article.

## Key Points

**Table:** 

Fruit-derived polyphenols have antioxidant and anti-inflammatory properties and so can enhance exercise performance, since excess reactive oxygen species generation has been implicated in fatigue development, and enhance recovery from intensive exercise due to the involvement of inflammation and oxidative damage within muscle.
Consumption of ~ 300 mg polyphenols an hour prior to exercise may enhance endurance and repeated sprint performance, most likely due to improved muscle perfusion.
Supplementation with > 1000 mg polyphenols per day for 3 or more days prior to and following exercise will enhance recovery from exercise-induced muscle damage.

## Introduction

Many sports and activities involve strenuous eccentric muscle contractions and explosive movements that can induce muscle damage to varying degrees depending on the intensity and duration of activity as well as the training status of the individual. Prolonged endurance events such as marathons and ultramarathons, triathlons and Ironman also place extreme demands upon muscle and can also induce ultrastructural damage and muscle soreness. It is therefore important to identify effective strategies for supporting rapid recovery between intensive training sessions especially during pre-season, when multiple sessions are completed in the same day, and between matches particularly in a tournament setting. The purpose of this paper is to provide an overview of the growing evidence that fruit-derived polyphenol supplementation enhances recovery from intensive exercise with or without muscle damage, and may enhance exercise performance.

## Polyphenols

Polyphenols are ubiquitous within plants, where they are produced as secondary metabolites and involved in a diverse range of critical processes including growth, pigmentation, pollination and resistance to pathogens and environmental stressors [1]. They are characterised structurally by two or more hydroxyl groups attached to one or more benzene rings, and can be classified into four main families: lignans, phenolic acids, stilbenes and flavonoids. Stilbenes consist of 2 × 6 carbon aromatic rings joined by a 2-carbon bridge with a double bond and the parent compound is resveratrol. Flavonoids consist of two aromatic rings linked through a 3-carbon chain, usually in the form of an oxygenated heterocycle; and can be sub-divided into a range of sub-classes on the basis of the degree of oxidation of the oxygenated heterocycle, including: flavanols, flavonols, flavanones, flavones, isoflavones and anthocyanidins. Tannins are oligomers and polymers of flavonoids, and can be grouped into condensed tannins (proanthocyanidins) and hydrolysable tannins (procyanidins). Table 1 provides a brief summary of example compounds and key dietary sources of the different polyphenol families. The taste and colour characteristics of fruits and vegetables are strongly influenced by the polyphenol content. Both the quantity and variety of polyphenols present are determined by the plant species, growing conditions sometimes termed terroir (sunlight, water and nutrient availability, temperature), post-harvest processing, and transport and storage conditions [2–4]. There is therefore considerable variability in the polyphenol content of foods on the supermarket shelves, and in the many polyphenol-rich fruit-derived supplements that are now commercially available. For research purposes, in particular, it is therefore essential that such natural variation can be accounted for by testing each batch for polyphenol content and composition.

**Table 1:** Dietary sources of the different polyphenol families and sub-families

Polyphenol family	Example compounds	Dietary source
Stilbenes	Resveratrol	Grapes
Lignans	Enterodiol	Seeds, whole grains, legumes
Phenolic acids	Cinnamic Benzoic	Caffeic acid–coffee Gallic acid–tea
Flavonoids Flavanols Flavonols Flavones Flavanones Isoflavones Anthocyanidins Proanthocyanidins Procyanidins	Epicatechin, Catechins Quercetin Luteolin Naringenin and hesperetins Genistein Cyanidin, malvidin, delphinidin B-type dimers Ellagitannins Gallotannins	Cocoa Green tea Onions, apples, deep green vegetables Parsley and other herbs Citrus fruits Soy products, Cherries and berries Cocoa Pomegranate Mango

Polyphenol absorption and metabolism is highly complex, not least because of the many thousands of different polyphenol compounds present within plants, their possible interactions within the food matrix [5] and their conjugation to form a large number of different metabolites upon absorption. In addition, with the exception of flavanols and proanthocyanidins, polyphenols are present as glycosides, i.e. conjugated with sugar moieties within the plant. Anthocyanins can be absorbed intact (e.g. cyanidin-3-glycoside), but many other polyphenols must be hydrolysed to split the sugar group (glycone) from the polyphenol (aglycone) before absorption [6]. This process occurs either via lactase phloridzin hydrolase in the brush border of the small intestine epithelial cells or via cytosolic β-glucosidase within the epithelial cells, which requires uptake of the polar glucosides into the epithelial cells via sodium-dependent glucose transporters [7]. The aglycones can then be absorbed into the circulation; however, they are subject to conjugation, resulting in the formation of sulfate, glucuronide and/or methylated metabolites both within the epithelial cells and in the liver [8]. A large proportion of polyphenols are not absorbed within the small intestine and continue to the colon where they are acted upon by enzymes present within the microbiota to release aglycones that then undergo ring fission to produce bioavailable metabolites such as phenolic acids and hydroxycinnamates [8]. These substances can be detected in the plasma some 12–48 h after polyphenol ingestion. Ingestion of a single polyphenol can give rise to many different metabolites, which was clearly shown in an elegant investigation where participants consumed 500 mg of ^13^C labelled cyanidin-3-glycoside, and 17 different labelled metabolites were detected in plasma over the subsequent 48 h [9]. Fruits and vegetables and fruit-derived polyphenol supplements contain a blend of polyphenols, and thus the pharmacokinetics and metabolism after ingestion of whole foods or fruit-derived supplements are even more complex. There is also a high degree of inter-individual variation in bioavailability partly dictated by differences in the gut microbiome [10]. To date the majority of studies that investigated the effects of polyphenol supplementation on either recovery from intensive exercise or exercise performance have not quantified plasma phenolic metabolites after supplementation, except for several studies employing cocoa or chocolate supplementation in which plasma epicatechin and catechin concentrations were measured [11, 12]. Whilst a number of published studies have measured acute changes in plasma phenolic metabolites or the parent compounds after single doses of Montmorency cherry [13], blueberry [14], blackcurrant [15] and pomegranate [16], future work in this field should ideally quantify exercise performance outcomes alongside measurement of plasma phenolic metabolites.

### Effects of Polyphenol Supplementation

Polyphenols possess radical scavenging properties related to their chemical structure. Phenolic hydroxyl groups can donate an electron to radicals and the polyphenol aromatic ring can then stabilise the resulting aroxyl radicals [17]. Polyphenols are also metal chelators and hence can reduce metal-catalysed free radical formation [18]. However, maximum plasma concentrations of conjugated and unconjugated polyphenols have been estimated to range from 0.1 to 22 µmol L^−1^ [19, 20]. When compared to the concentration of plasma urate (150–450 µmol L^−1^), which is also an important plasma compartment antioxidant, it seems unlikely that plasma phenolics are effective direct antioxidants in vivo. Many of the studies reviewed in this article have quantified changes in the debunked [21] plasma total antioxidant capacity (TAC) after polyphenol supplementation. However, TAC only quantifies the cumulative action of small molecule antioxidants present in plasma, and ignores the important contribution of intracellular enzymes such as superoxide dismutase, catalase and peroxiredoxin. This cannot therefore be regarded as a true measure of in vivo antioxidant capacity. The increases in TAC observed after polyphenol supplementation are likely to be almost entirely dependent upon changes in plasma urate related to fructose metabolism, and independent of changes in plasma phenolics [22]. There is now growing evidence that phenolics are able to upregulate endogenous antioxidant capacity via the Nrf2/antioxidant response element (ARE) pathway. Nrf2 is a master regulator of the antioxidant response through the regulation of a wide range of antioxidant and phase II detoxification genes [23], and protects cells from stressors including reactive oxygen species (ROS), and dietary xenobiotics such as polyphenols [24]. Keap1 is a cysteine-rich protein that represses Nrf2 signalling by serving as a bridge between Nrf2 and ubiquitination ligase cullin-3, which is required for the ubiquitination of the protein and subsequent proteasomal degradation [25, 26]. Oxidative stressors or electrophiles induce covalent modification of Keap1 cysteine residues and therefore inhibit ubiquitination-dependent degradation and increase nuclear accumulation of Nrf2, resulting in increased synthesis of downstream endogenous antioxidants such as superoxide dismutase, catalase and peroxiredoxin [27]. Although dietary polyphenols are not present in sufficient quantity in vivo to contribute directly to antioxidant function as radical scavengers, phenolics will be converted to electrophilic quinones and hydroquinones upon exposure to ROS, which are then able to interact with Keap 1 and activate Nrf2 (for a review, see Huang et al. [24]). Paradoxically it seems that antioxidant effects of polyphenols arise from their pro-oxidant action after in vivo exposure to ROS, although much of the evidence for this is currently from in vitro work, and there is a need for human in vivo studies to verify this hypothesis. Polyphenols also seem to possess anti-inflammatory properties and have been shown in vitro to inhibit cyclo-oxygenase enzymes, COX1 and COX2 [28], to reduce NF-ƘB expression signalling [29] and to inhibit nuclear translocation of NF-ƘB [30].

In resting human studies, changes in plasma biomarkers of oxidative damage and inflammation support the antioxidant and anti-inflammatory effects of fruit-derived polyphenols including cherries [31], blueberries [32, 33], blackcurrant [34], pomegranate [35] and cocoa [36]. There is also consistent evidence that both acute and chronic supplementation with polyphenols improves vascular function, specifically endothelium-dependent vasodilation, which is nitric oxide (NO) dependent and measured in vivo as flow-mediated dilatation (FMD). A meta-analysis of 42 cocoa studies found significant increases in FMD following acute (3.2%) and chronic (1.3%) supplementation, respectively [37]. A smaller number of studies have been performed to assess the acute or chronic effects of fruit polyphenols on FMD, and these have mostly utilised grape-derived polyphenols as well as pomegranate, chokeberry or blueberry. Rodriguez-Mateos et al. [14] showed that a range of blueberry polyphenol doses (0.3–1.88 g polyphenols) increased FMD in healthy men peaking at 1 h and this paralleled reduction in neutrophil nicotinamide adenine dinucleotide phosphate (NADPH) oxidase activity. This suggests that reduced superoxide production and hence decreased conversion of NO to peroxynitrite likely contributes to the increased vasodilation response by improving NO bioavailability.

A number of studies have also found improvements in endothelial-dependent vasodilatation after chronic supplementation with fruit polyphenols especially amongst study populations with impaired cardiovascular function (grape juice [38, 39], red grapes [40], grape polyphenols [41], grape seed [42], chokeberry juice [43]) or with low habitual fruit and vegetable intake (blackcurrant [44]). A meta-analysis revealed that after supplementation with a mix of flavonoids, the absolute mean difference in % dilatation was ∆ 2.33% (based on 18 acute supplementation studies) and ∆ 0.73% with chronic supplementation (based on 14 studies) [45]. The optimal dose identified was 500 mg per day total flavonoids or 300 mg per day of procyanidins.

The improvement in FMD in response to acute and chronic polyphenol supplementation is by definition a result of increased NO bioavailability, since FMD is NO dependent. These effects are likely achieved through a variety of synergistic mechanisms. There is evidence from in vitro studies that polyphenols induce endothelial nitric oxide synthase 3 (eNOS) activation via signalling through oestrogen receptor-α via G protein, extracellular-signal-regulated kinase (ERK) and phosphatidylinositide 3-kinase (PI3 K) pathways [46]. In addition, polyphenols have been shown to inhibit NADPH oxidase, one of the key sources of superoxide production [47], and to induce signalling through Nrf2, thus increasing endogenous antioxidant capacity [48], both of which will preserve NO bioavailability by reduced formation of peroxynitrite. In addition to the health benefits associated with these antioxidant, anti-inflammatory and vasoactive properties of polyphenols, they are also of relevance for exercise performance and recovery from intensive exercise.

## Fruit-derived Polyphenols and Exercise Performance

### Rationale

Exercise-induced fatigue has been extensively investigated, but the mechanisms remain controversial due its complex multifactorial nature and specificity to exercise mode, intensity and duration. Fatigue may relate to: (i) depletion of muscle fuel substrates such as phosphocreatine (PCr) or glycogen, (ii) accumulation of metabolic by-products such as ammonia and hydrogen ions, (iii) perturbation of ionic gradients between intra- and extracellular compartments thus interfering with action potential transmission, (iv) impaired excitation contraction coupling via altered calcium sensitivity and sequestration to the sarcoplasmic reticulum, (v) altered central drive due to afferent feedback from the exercising muscle conveying changes in the metabolic milieu (for a review, see Allen et al. [49]). The ability to regulate and co-ordinate the function of cardiovascular, respiratory, metabolic and neuromuscular systems is pivotal to ensure that the kinetics of substrate supply and waste product removal match the requirements of the specific exercise bout and thus performance is preserved.

Skeletal muscle is a net producer of reactive oxygen species from a variety of sources including the mitochondrial respiratory chain and enzymatic sources such as NADPH oxidase and xanthine oxidase [50]. Paramagnetic electron spin resonance experiments demonstrate that ROS generation increases with exercise intensity [51, 52]. ROS are important signalling molecules and have been implicated in contraction-mediated increases in muscle glucose uptake [53] and control of skeletal muscle blood flow [54]. For instance, Durand et al. [55] found that hydrogen peroxide caused vasodilation in exercising muscle. It appears that under conditions of low oxidative stress and redox balance, ROS promote optimal vasodilation and hyperaemia in exercising muscle. However, under conditions of oxidative stress or already disturbed redox balance, ROS generation during exercise impairs blood flow and vasodilatory capacity [56]. Ryanodine receptors are the major calcium release channel in the sarcoplasmic reticulum (SR), and due to the high number of cysteine residues in this protein, it is redox sensitive [57], as is SR Ca2 + ATPase activity. Excess ROS generation has been shown to impair calcium handling and sensitivity resulting in reduced contractile force development, thus impairing exercise performance [50, 58]. Additionally, group III and IV afferents are sensitive to ROS, so ROS production within exercising muscle will be sensed by the somatosensory cortex, which may result in altered efferent activity and central drive to the muscle, contributing to the development of central fatigue [59]. Therefore, during high intensity or prolonged exercise where ROS generation exceeds the antioxidant capacity and results in disturbed redox balance, it is plausible that antioxidant supplementation may counteract fatigue and enhance performance via enhanced perfusion of the exercising muscle, better maintained excitation–contraction coupling and central drive. There is some empirical evidence to support this view, since supplementation with antioxidants such as *N*-acetyl cysteine [60–62] has been shown to improve exercise performance. There is also evidence that acute (Table 2) or chronic (Table 3) supplementation with fruit-derived polyphenols is ergogenic for performance.

**Table 2:** Effects of acute polyphenol supplementation on exercise performance

References	Participant characteristics	Supplementation protocol	Performance task	Effects of polyphenol supplementation
Cases et al. [66]	Recreationally active men (*n* = 15) Crossover trial	290 mg PP from green tea, grape and pomegranate 60 min pre-ex	4 × 30 s all-out cycle sprints with 4-5 min inter-sprint recovery	↑ Peak and average power output ↑ Erythrocyte catalase and plasma SOD activity ↓ Post-exercise pulse pressure
Crum et al. [64]	Trained cyclists (*n* = 8) Crossover trial	1000 mg pomegranate extract 2.5 h pre-ex (low PP diet consumed)	Time to exhaustion at 100% ${\dot{\text V}}{\text O}_{2}\max$ workload at sea level and 1657 m	= Time to exhaustion ↑ Plasma nitrate concentration ↑ ${\dot{\text V}}{\text O}_{2}$ at altitude
Roelofs et al. [65]	Recreationally resistance trained (*n* = 19) Crossover trial	1000 mg pomegranate extract 30 min pre-ex	10 × 6 s cycle sprints 30 s recovery Bench press and leg press repetitions to failure @ 80% 1RM	= Average and peak power (CI revealed higher peak s5 and 7 and average s5 power) = Bench press and leg press repetitions ↑ Brachial artery diameter and flow after repetitions to failure and tendency after repeated sprints.
Trexler et al. [63]	Recreationally active (*n* = 19) Crossover trial	1000 mg pomegranate extract 30 min pre-ex	Treadmill runs to exhaustion at 90, 100, 110% ${\dot{\text V}}{\text O}_{2}\max$ (PV)	↑ Time to exhaustion at 90% and 100% PV ↑ Brachial artery blood flow pre-ex ↑ Brachial artery diameter post-ex
Decroix et al. [11]	Trained male cyclists (*n* = 12) Crossover trial	900 mg cocoa flavanols 1.5 h (TT1) and 3 h (TT2) pre-ex (low PP diet consumed 24 h pre-ex)	Two cycling time trial (equivalent of 30 min @ 75% peak power output) with 100 min passive recovery	= Time trial performance (+ve trend for TT1, *p* = 0.09) ↑ Total antioxidant capacity (corrected for ↑ uric acid) = Plasma marker oxidative stress (malondialdehyde) = Plasma inflammatory markers (IL1, IL6 and TNF-α)
Deley et al. [69]	Recreationally active men RCT	500 mg PP from grape and apple (*n* = 24) versus placebo (*n* = 24) Prior evening and 1 h pre-ex	Time to exhaustion at 70% maximum cycling aerobic power	↑ Time to exhaustion (9.7%)
Oh et al. [68]	Recreationally active men (*n* = 20) Crossover trial	72 mg PP from Ecklonia cava 30 min pre-ex	Incremental maximum treadmill test	↑ Time to exhaustion Non-significant improvement in ${\dot{\text V}}{\text O}_{2}\max$ (6.5%)
Keane et al. [67]	Trained cyclists (*n* = 10) Crossover trial	60 ml Montmorency cherry concentrate (PP dose not provided) 1.5 h pre-ex	6 min moderate and 6 min severe intensity cycling continued to exhaustion and on other occasion followed by an all-out 60 s sprint	= Time to exhaustion ↑ Peak power and work done during sprint ↓ Pre-ex systolic blood pressure

↑ Increased, ↓ decreased, = no change, *CI* confidence interval, *IL* interleukin, *PP* polyphenol, *PV* peak velocity, *RCT* randomised controlled trial with parallel groups, *RM* repetition maximum, *SOD* superoxide dismutase, *TNF* tumour necrosis factor, *TT* time trial, ${\dot{V}}{O}_{2}max$ maximum rate of oxygen consumption

**Table 3:** Effects of chronic polyphenol supplementation on exercise performance

References	Participant characteristics	Supplementation protocol	Performance task	Effects of polyphenol supplementation
Braakhuis et al. [80]	Trained female runners (*n* = 23) Crossover trial	Vitamin C (0.5 g), blackcurrant (7.5 mg vitamin C and 150 mg anthocyanins), placebo × 2 per day for 3 weeks (low PP diet consumed on days of blood tests)	3-week blocks of intensified training 5 km running time trial and maximum incremental treadmill test pre/post each block	= Performance (possible benefit of blackcurrant for faster runners) = Plasma and erythrocyte markers of oxidative stress (in contrast to vitamin C which exerted pro-oxidant effects: ↑SOD, CAT and PC)
Cook et al. [75]	Recreationally active men (*n* = 14) Crossover trial	300 mg blackcurrant per day (105 mg anthocyanins) for 7 days	16.1 km cycling time trial after 30 min steady state cycling	↑ Time trial performance (2.4%) ↑ Fat oxidation during SS cycling (indirect calorimetry 27%) ↑ Blood lactate concentration (~ 12%)
Godwin et al. [78]	Recreationally active University players (*n* = 15) and trained youth (*n* = 9) male footballers. Crossover trial	300 mg blackcurrant per day (105 mg anthocyanins) for 7 days	6 × 35 m sprints interspersed with 10 s passive recovery	Trend for improved fatigue index (~ 12%, *p* = 0.06)
Murphy et al. [79]	Trained male cyclists (*n* = 10) Crossover trial	300 mg blackcurrant per day (105 mg anthocyanins) for 7 days	2 × 4 km time trials interspersed with 10 min active recovery	↑ Performance (0.8%) = Blood lactate concentration and heart rate
Perkins et al. [76]	Recreationally active men (*n* = 13) Crossover trial	300 mg blackcurrant per day (105 mg anthocyanins) for 7 days	High intensity intermittent treadmill running to exhaustion (stages of sprints 6 × 19 s and 15 s low intensity running)	↑ Running distance (~ 10%) = HR, ${\dot{\text V}}{\text O}_{2}\max$, blood lactate, RPE for first 4 stages Trend for ↑ blood lactate concentration at exhaustion (*p* = 0.07)
Willems et al. [77]	Recreationally active men (*n* = 13) Crossover trial	300 mg blackcurrant per day (105 mg anthocyanins) for 7 days	Loughborough Intermittent Shuttle Test	= Time to exhaustion ↓ Slowing of the fastest maximal sprint across blocks
Allgrove et al. [82]	Recreationally active men (*n* = 20) Crossover trial	40 g dark chocolate (54 mg catechins and 44 mg flavanols) twice per day for 2 weeks (last dose 2 h pre-ex) (low PP diet consumed 48 h pre-ex)	Pre-load: 60% ${\dot{\text V}}{\text O}_{2}\max$ for 90 min with 30 s at 90% ${\dot{\text V}}{\text O}_{2}\max$ every 10 min Time to exhaustion at 90% ${\dot{\text V}}{\text O}_{2}\max$	= Time to exhaustion ↑ Plasma free fatty acid concentration ↓ Plasma markers of oxidative stress (F2-isoprostanes, oxidised low density lipoproteins) = Plasma markers of inflammation
Kang et al. [73]	Recreationally active RCT	100 mg oligomerized lychee extract (*n* = 24), or vitamin C (400 mg) and E (160 IU) (*n* = 24), or placebo (*n* = 22) for 30 days (no fruit/vegetables for 3d pre-ex)	Time to exhaustion treadmill running at 80% HRmax	↑ Time to exhaustion = ${\dot{\text V}}{\text O}_{2}\max$
Richards et al. [83]	Recreationally active (*n *= 19) Crossover trial	135 mg epigallocatechin × 3 per day for 2 days and 2 h pre-ex	Incremental maximum cycle exercise test	↑ ${\dot{\text V}}{\text O}_{2}\max$ = Maximum cardiac output
Sadowska-Krepa et al. [74]	Recreationally active RCT	73 mg PP from 390 mg red grape skin extract (*n* = 9) vs placebo (*n* = 5) × 3 per day for 6 weeks	6 × 50 m swimming time trials	↑ Performance (faster speed, = heart rate) = Plasma total antioxidant capacity and antioxidant enzyme activity (e.g. glutathione reductase, catalase, superoxide dismutase) ↓ Blood creatine kinase activity
Trinity et al. [81]	Trained male cyclists (*n* = 12) Crossover trial	500 ml pomegranate or placebo × 2 per day for 7 days (~ 1800 mg PP per day)	10 min cycling time trial after 50 min pre-load time to exhaustion at ${\dot{\text V}}{\text O}_{2}\max$ power output All at 31.5 °C and 55% humidity	= Time trial performance = Time to exhaustion = Heart rate, stroke volume, cardiac output

↑ Increased, ↓ decreased, = no change, *CAT* catalase, *ex* exercise, *HR* heart rate, *HRmax* maximal heart rate, *PC* protein carbonyls, *PP* polyphenol, *RCT* randomised controlled trial with parallel groups, *RPE* rating of perceived exertion, *SOD* superoxide dismutase, *SS* steady state, ${\dot{V}}$*O*_*2*_*max* maximum rate of oxygen consumption

### Evidence of Enhanced Performance

#### Acute Polyphenol Supplementation

Pomegranate has been a key ingredient in the majority of the relatively small number of studies in which the effects of acute polyphenol supplementation (single dose < 3 h pre-exercise) have been investigated either consumed singly [63–65] or in combination with green tea and grape polyphenols [66]. Consumption of 1 g of pomegranate extract [63] 30 min pre-exercise or a combined supplement of pomegranate, green tea and grape extract (290 mg polyphenols [66]) consumed 1 h pre-exercise enhanced time to exhaustion whilst running at 90 and 100% of peak velocity achieved at ${\dot{\text V}}{\text O}_{2}\max,$ and peak and average power output during repeated cycle Wingate tests in recreationally active individuals, respectively. However, trained cyclists [64] and recreationally resistance-trained individuals [65] did not derive significant performance benefit from consumption of 1 g of pomegranate extract. Specifically, time to exhaustion at 100% ${\dot{\text V}}{\text O}_{2}\max$ workload was not enhanced by consumption of pomegranate 2.5 h pre-exercise either at sea level or at the equivalent of 1657 m altitude, despite a significant increase in oxygen consumption in the altitude condition after supplementation, and increased plasma nitrate concentration after pomegranate supplementation [64]. Similarly, bench and leg press repetitions to failure and average and peak power during 10 × 6 s sprints were not significantly elevated by pomegranate supplementation (30 min pre-exercise), although confidence interval-based statistics indicated increased power output during the middle sprints, (S5 and 7 [65]) despite evidence of favourable vascular effects after supplementation, with elevated brachial artery diameter and flow post-exercise in the pomegranate condition [65]. Other polyphenol-containing supplements have also shown mixed results when used prior to exercise in an attempt to improve performance. Trained cyclists experienced modest improvements in end-sprint performance 1.5 h after ingestion of Montmorency cherry concentrate [67]. Recreationally active men also apparently derived ergogenic effects from supplementation with ecklonia cava extract [68] and a combined grape and apple polyphenol supplement [69], with increased time to exhaustion during a maximal incremental treadmill running test, and cycling at 70% maximum aerobic power, respectively. Conversely, consumption of 900 mg cocoa flavanols 3 h pre-exercise did not significantly enhance cycling time trial performance in trained male cyclists [11].

Analysis of the limited published data currently available suggests that timing of consumption may also be an important factor, since in those studies where supplements were consumed within 1 h of exercise, ergogenic effects were evident, but where the supplement was consumed > 2 h prior to exercise, performance was not improved. Unfortunately, plasma phenolic concentrations were not measured in these studies; however, extrapolation from other studies (blueberry [70], Montmorency cherry [13], pomegranate [16], grape [71] and cocoa flavanols [72]) would suggest that elevations in plasma phenolics would be evident within 30 min of consumption with peak concentrations of simple phenolic metabolites likely within 1–2 h of ingestion. These changes presumably underpin the observed ergogenic effects, which are likely to involve inhibition of superoxide-producing enzymes such as NADPH oxidase [14], enhanced antioxidant enzyme activity [66] and vascular mechanisms ([63, 65, 66], see Sect. 3.3).

#### Chronic Polyphenol Supplementation

Chronic polyphenol consumption also seems to produce ergogenic effects for recreationally active participants and to a lesser extent for trained athletes. Consumption of oligomerized lychee extract for 30 days (200 mg polyphenols) enhanced running time to exhaustion at 80% heart rate maximum [73], and red grape skin extract for 6 weeks (1.17 g·day^−1^, 220 mg polyphenols [74]) improved 50-m swimming time trial performance in recreationally active participants. Blackcurrant powder (300, 105 mg anthocyanins) consumption for 7 days (~ 105 mg anthocyanins) enhanced cycling time trial performance (16.1 km [75]) and high-intensity intermittent running distance to exhaustion [76], and reduced fatigue index with repeated sprints in recreationally active participants [77, 78], and in trained individuals induced a small (0.8%) improvement in repeated 4-km cycling time trial performance [79]. Braakhuis et al. [80] also found a possible improvement in 5-km time trial performance for faster female runners after 3 weeks’ blackcurrant polyphenol supplementation (300 mg anthocyanins) during a period of intensified training, with evidence of performance decrement with vitamin C supplementation in this randomised crossover trial. However, 7 days of pomegranate supplementation (1800 mg polyphenols) did not enhance cycling time trial performance in trained male cyclists [81]. Nor did 2 weeks of dark chocolate supplementation (108 mg catechins and 88 mg flavanols) enhance time to exhaustion at 90% ${\dot{\text V}}{\text O}_{2}\max$ in recreationally active men, despite elevated plasma free fatty acid concentrations and evidence of reduced exercise-induced oxidative damage (F2-isoprostanes and oxidised low-density lipoprotein cholesterol [82]).

For many of the studies it is not clear how long after the last polyphenol dose exercise was performed. In other words, it is hard to differentiate a chronic effect from the potential acute effects derived from supplementation within 2 h of exercise. All reported studies involved supplementation for ≥ 7 days, which should provide sufficient time for changes in gene and protein expression to occur within tissue. However, to our knowledge there are currently no data available from studies that combine measurement of changes in muscle proteins in response to polyphenol supplementation with changes in exercise performance. From the available evidence, the most likely mechanisms appear to be reduced exposure to or increased capacity to degrade ROS and reactive nitrogen species (RNS) as evidenced by reduced markers of oxidative damage [80, 82] or increased antioxidant enzyme activity [74]. These responses seem to occur in parallel with enhanced vascular function possibly resulting in improved muscle perfusion and enhanced oxygen extraction [83].

### Mechanisms

The conflicting findings across studies with regard to the effects of polyphenol supplementation are most likely due to the differences in research design including: the supplement composition, timing and dose of supplementation variation in the studied population, and performance tasks employed and therefore differences in the predominant processes contributing to fatigue development. The ergogenic effects of polyphenols seem to involve altered vascular function with augmentation of the measured increases in brachial artery diameter and blood flow after a 1 min occlusion, induced by pomegranate supplementation, which paralleled the ergogenic effects for performance [63, 65]. Similarly, Richards et al. [83] found that ${\dot{\text V}}{\text O}_{2}\max$ was enhanced by supplementation with green tea-derived epigallocatechin (EGCG) supplementation for 2 days (135 mg three times per day) and a last dose 2 h pre-exercise. However, maximum cardiac output was not affected by EGCG, which therefore implies that the performance-enhancing effects were achieved via increased arterio-venous oxygen difference, i.e. improved oxygen extraction in the exercising muscle presumably coupled with better spatial distribution of muscle perfusion. Goncalves et al. [84] used nail-fold videocapillaroscopy of the fourth finger of the left hand in a temperature-controlled environment to assess microvascular function in triathletes in response to consuming 300 ml·day^−1^ of organic grape juice (~ 1600 mg polyphenols) for 20 days. Functional capillary density (capillaries with red cell flux), red blood cell velocity and time to peak velocity after 1 min arterial occlusion were all improved after grape juice supplementation, supporting the likelihood of better tissue perfusion after polyphenol supplementation, although this clearly needs to be directly tested in muscle. The ergogenic effects of blackcurrant supplementation are also proposed to be dependent on vascular mechanisms, but until recently there has been limited empirical data to support this assertion. Cook et al. [85] found that 7 days of supplementation with 600 mg of blackcurrant powder (twice the daily dose provided in the exercise performance studies [75–77, 79]) resulted in increased femoral artery diameter during a sustained submaximal maximum voluntary contraction (MVC) of the knee extensors (30% MVC for 120 s). These effects were accompanied by lower systolic and diastolic blood pressure and lower total peripheral resistance estimated via beat–beat finger blood pressure analysis.

The most plausible mechanisms for these apparent vascular effects are either reduced generation of ROS or improved capacity to detoxify ROS via antioxidant systems. Such reduced exposure to ROS will improve bioavailability of the potent vasodilator NO, due to reduced production of peroxynitrite via the reaction of superoxide and NO. In vivo and in vitro evidence demonstrate that phenolic metabolites reduce NADPH oxidase activity, one of the key sources of superoxide production during exercise [47]. This is corroborated by the evidence that acute polyphenol supplementation protects against endurance exercise-induced oxidative damage. Consumption of a black grape, raspberry and redcurrant polyphenol blend (2000 mg polyphenols, including 1212 mg anthocyanins) during 90 min of cycling at 70% ${\dot{\text V}}{\text O}_{2}\max$ attenuated the exercise-induced elevation in plasma thiobarbituric acid-reacting substances (TBARS) and protein carbonyls in trained male cyclists [86]. Recreationally active participants experienced a reduced F2-isoprostane response to 2.5 h cycling at 60% ${\dot{\text V}}{\text O}_{2}\max$ when completed 2 h after consuming 100 g of dark chocolate (~ 250 mg polyphenols [12]). Lyall et al. [87] found that the increase in plasma protein carbonyls after 30 min rowing at 80% ${\dot{\text V}}{\text O}_{2}\max$ was attenuated when blackcurrant powder (120 mg anthocyanins) was consumed immediately prior to exercise by recreationally active participants. However, none of these studies included an assessment of exercise performance.

In addition, there is evidence that chronic polyphenol consumption increases endogenous antioxidant system capacity via signalling through Nrf2 and antioxidant response element pathways (see Sect. 2) in a similar fashion to exercise adaptation [88]. Chronic polyphenol supplementation lowers exercise-induced oxidative stress, for instance 250 g blueberries consumed every day for 6 weeks with 375 g consumed 2 h prior to a 2.5 h run at 72% ${\dot{\text V}}{\text O}_{2}\max$ attenuated the exercise-induced increase in plasma F2-isoprostanes and urinary oxidation products of RNA [89]. Young men consuming a diet supplemented with anthocyanin-rich purple sweet potato leaves versus a low polyphenol diet for 7 days experienced reduced plasma protein carbonyls and TBARS at rest and after 1 h exercise at 70% ${\dot{\text V}}{\text O}_{2}\max$ [90]. Fuster-Munoz et al. [91] found that plasma malondialdehyde (MDA) and PC were reduced in endurance athletes undergoing normal training when consuming 200 ml·day^−1^ of pomegranate juice for 21 days. The ergogenic effects of polyphenols therefore seem to be underpinned by vascular and antioxidant mechanisms. However, there is clearly a need for well-designed studies that measure exercise performance alongside robust measures of oxidative damage, antioxidant enzyme content and activity, inflammatory processes, macro- and micro-vascular function, and plasma phenolics to confirm these ergogenic effects and the mediators of these effects.

## Fruit-derived Polyphenols and Recovery after Intensive Exercise

### Rationale

Exercise-induced muscle damage involves both mechanical and biochemical processes. Muscle fibres are exposed to high mechanical forces especially during explosive and/or eccentric contractions, which can result in disruption to the extracellular matrix, contractile proteins, sarcoplasmic reticulum, t-tubules and sarcolemma. There is subsequent disruption of calcium homeostasis with elevated resting calcium concentration [92], which may then contribute to further damage through activation of calcium sensitive proteases such as calpain resulting in increased proteolysis of susceptible proteins such as desmin and actin, thus worsening the ultrastructural damage [92]. In addition, there is increased generation of reactive oxygen species in an exercise intensity- and mode-dependent fashion [51, 93], which will cause oxidative modification of proteins and other molecules resulting in altered function and damage. Such processes will be particularly important contributing factors to the initial damage induced by high-intensity prolonged activities that do not involve eccentric muscle actions.

This initial damage, whether mechanical or biochemical, triggers a potent inflammatory response with damaged fibres releasing pro-inflammatory cytokines, which serve as chemo-attractants for neutrophils and macrophages and activate ROS generating enzymes within the muscle [94]. Neutrophil infiltration and activation occurs within 2 h of damage, generally peaking within 6–24 h and then rapidly decreasing [95]. Neutrophils are rich in enzymes such as NADPH oxidase, and release ROS and proteolytic enzymes that may exacerbate the initial muscle damage [96], but also facilitate regeneration by removal of debris and activation of satellite cells [97]. Shortly after neutrophil infiltration, macrophages derived from blood monocytes accumulate within the damaged tissue. Their role is to scavenge debris and apoptotic cells and in addition release a range of growth factors and other substances that trigger remodelling of extracellular matrix, contractile and vascular elements. Two macrophage populations have been identified: M1 and M2. M1 macrophages are pro-inflammatory and infiltrate muscle in the early stages of damage, and have been linked to proliferation of satellite cells [98]. It has been suggested that phagocytosis of damaged myogenic cells by M1 macrophages triggers their conversion to M2 macrophages [99]. These M2 macrophages release anti-inflammatory cytokines (transforming growth factor-β and interleukin 10) and release growth factors such as insulin-like growth factor 1 to support regeneration of the damaged tissue [100].

The central involvement of ROS generation and inflammation within the muscle damage and healing process suggests that polyphenol supplementation will influence these processes and therefore the rate of recovery. Polyphenols have been shown to inhibit activity of key ROS-generating enzymes such as NADPH oxidase, serve as radical scavengers, and with chronic supplementation also enhance endogenous antioxidant capacity. In addition, polyphenols have been shown to inhibit cyclo-oxygenase activity and suppress inflammation [101–103].

### Evidence of Enhanced Functional Recovery

There is now a growing body of evidence that suggests that fruit-derived polyphenol supplementation enhances restoration of muscle function and reduces soreness after intensive exercise (Table 4). The effects of Montmorency cherry supplementation on recovery of muscle function or exercise performance after intensive exercise have been investigated in nine published studies thus far, of which five studies found favourable effects [104–108]. These studies involved recreationally active men [104], recreational runners [105] or trained athletes [106–108], so unlike the acute performance effects of polyphenol supplementation, it seems that beneficial effects on recovery are accessible to both trained and less well-trained individuals. Nor does the efficacy of the supplement seem to be influenced by the mode of exercise used to induce muscle damage, since different approaches or muscle groups were damaged in each of these studies (intensive knee extensor resistance exercise [106]; eccentric elbow flexor exercise [104]; marathon running [105]; high-intensity stochastic cycling [108] or 90 min repeated high-intensity shuttle running [107]). In these studies, Montmorency cherry was provided in the form of a juice drink, which was consumed morning and evening for at least 3 days prior to exercise, and provided at least 1200 mg polyphenols per day. Conversely, in studies where recovery of muscle function or exercise performance was not enhanced [109–111, 120], either a lower (and presumably insufficient) dose of Montmorency cherry was provided in powder form (480 mg Montmorency cherry powder in a single dose [109]; 733 mg polyphenols split into two doses [110]) or the intensive exercise task did not induce a measurable decline in muscle strength [110] or exercise performance [111], thus by definition making it impossible to improve recovery. As well as differences in dose and frequency of supplementation, it is possible that differences in post-harvest processing of cherries between juice and powder production may induce variation in the polyphenol blend, and thus contribute to the discrepancy in findings across studies utilising Montmorency cherry powder [109, 110] versus juice [104–108, 111, 112]. In summary, consumption of Montmorency cherry juice or concentrate providing 600 mg polyphenols morning and evening for at least 3 days prior to exercise and during recovery has consistently been shown to improve recovery of muscle function. Further studies are required to identify the optimal dose, frequency and duration of consumption.

**Table 4:** Effects of fruit-derived polyphenol supplementation on recovery from exercise-induced muscle damage

References	Participant characteristics	Supplementation protocol	Muscle damage protocol	Effects of polyphenol supplementation
Beals et al. [110]	Recreationally active RCT	30 g tart cherry powder (*n* = 15) versus placebo (*n* = 15) × 2 per day for 12 days (733 mg PP, 64 mg anthocyanins)	Maximal effort unilateral isokinetic concentric/eccentric knee extensor contractions of at 60^o^·s^−1^ until 50% drop in force Performed on d5 supplementation	= Isometric knee extensor muscle force = Muscle soreness = Plasma biomarkers of inflammation
Bell et al. [120]	Trained cyclists RCT	30 ml Montmorency cherry concentrate (600 mg PP, 254 mg anthocyanins) (*n* = 8) versus placebo (*n* = 8) × 2 per day for 7 days (low PP diet consumed throughout trial)	High-intensity stochastic cycling exercise (109 min) Performed on days 5, 6, 7	↓ Plasma biomarkers of inflammation (IL6 and CRP) ↓ Plasma marker of oxidative damage (lipid hydroperoxides)
Bell et al. [108]	Trained cyclists RCT	30 ml Montmorency cherry concentrate (600 mg PP, 254 mg anthocyanins) (*n* = 8) versus placebo (*n* = 8) × 2 per day for 7 days (low PP diet consumed throughout trial)	High intensity stochastic cycling exercise (109 min) Performed on days 5	↑ isometric knee extensor muscle force, cycling economy ↓ plasma markers of inflammation (IL6 and CRP) = plasma marker of damage (CK and lipid hydroperoxides)
Bell et al. [107]	Semi-professional male soccer players RCT	30 ml Montmorency cherry concentrate (600 mg PP, 254 mg anthocyanins) (*n* = 8) versus placebo (*n* = 8) × 2 per day for 8 days (low PP diet consumed throughout trial)	Loughborough Intermittent Shuttle Test Performed on d5 supplementation	↑ Isometric knee extensor muscle force, countermovement jump height, and agility ↓ Muscle soreness ↓ Plasma markers of inflammation (IL6) = Plasma markers of damage (CK and lipid hydroperoxides)
Bowtell et al. [106]	Trained high intensity intermittent sport athletes (*n* = 10) Crossover trial	30 ml Montmorency cherry concentrate (600 mg PP, 254 mg anthocyanins) × 2 per day for 10 days	10 sets of 10 unilateral knee extension repetitions @ 80% 1RM Performed on d8 supplementation	↑ Isometric knee extensor muscle force = Muscle soreness and CK = Plasma biomarkers of inflammation (CRP) ↓ Plasma marker of oxidative damage (PC)
Connolly et al. [104]	Recreationally active men (*n* = 14) Crossover trial	12 oz bottles Montmorency cherry concentrate in apple juice (600 mg PP, incl 40 mg anthocyanins) × 2 per day for 8 days	2 × 20 maximum unilateral eccentric elbow flexor contractions Performed on d4 supplementation	↑ Isometric elbow flexor muscle force ↓ Muscle soreness = Muscle tenderness and relaxed elbow joint angle
Howatson et al. [105]	Recreational marathon runners (*n* = 20) RCT	8 oz bottles Montmorency cherry juice (600 mg PP, incl 40 mg anthocyanins) (*n* = 10) versus placebo (*n* = 10) × 2 per day for 8 days	Marathon Performed on d6 supplementation	↑ Isometric knee extensor muscle force = Muscle soreness, CK and LDH ↓ Plasma biomarkers of inflammation (IL6 and CRP) Plasma markers of oxidative damage (↓TBARS, = PC)
Kuehl et al. [112]	Recreational runners RCT	10.5 oz Montmorency cherry juice (600 mg PP, incl 40 mg anthocyanins) (*n* = 28) versus placebo (*n* = 26) × 2 per day for 8 days	Hood to Coast relay race 26.3 ± 2.5 km distance Performed on d8 supplementation	↓ Muscle soreness
Levers et al. [109]	Resistance trained men RCT	480 mg Montmorency cherry powder (*n* = 11) versus placebo (*n* = 12) × 1 per day for 10 days	10 sets of 10 repetitions @ 70% 1RM barbell back squats Performed on d8 supplementation	= Isometric knee extensor muscle force ↑ Pressure pain tolerance = Plasma biomarkers of inflammation = Plasma marker of oxidative damage
Machin et al. [116]	Recreationally active men RCT	30 ml pomegranate concentrate (650 mg PP, 620 mg ellagitanins) × 1 (*n* = 15) or × 2 (*n* = 15) per day or placebo (*n* = 15) for 8 days	3 × 20 maximum unilateral eccentric elbow flexor contractions 20 min downhill running Performed on d4 supplementation	↑ Isometric elbow flexor and knee extensor strength = Muscle soreness = Blood muscle damage markers (Mb)
McCormick et al. [111]	Trained waterpolo players (*n* = 9) Crossover trial	30 ml Montmorency cherry concentrate (600 mg PP, 254 mg anthocyanins) × 3 per day for 7 days	Fatiguing simulated water polo team activity Performed on d6 supplementation	= Performance waterpolo specific tasks = Plasma biomarkers of inflammation and oxidative damage
Trombold et al. [114]	Recreationally active men (*n* = 16) Crossover trial	500 ml pomegranate juice (650 mg PP, 620 mg ellagitanins) × 2 per day for 9 days	2 × 20 maximum eccentric elbow flexor contractions Performed on d5 supplementation	↑ Isometric strength = Muscle soreness = Muscle damage (CK, Mb) = Plasma markers of inflammation (IL6, CRP)
Trombold et al. [115]	Resistance trained men (*n* = 17) Crossover trial	250 ml pomegranate juice (495 mg tannins, 96 mg anthocyanins, 30 mg ellagic acid derivatives) × 2 per day for 15 days	3 × 20 maximum unilateral eccentric elbow flexor contractions 6 × 10 unilateral maximum knee extensor contractions Performed on d8 supplementation	↑ Isometric elbow flexor strength ↓ Elbow flexor muscle soreness = Isometric knee extensor strength = Knee extensor muscle soreness
Hutchison et al. [35]	Untrained RCT	12 oz blackcurrant juice (369 mg anthocyanins) (*n* = 8) versus placebo (*n* = 8) × 2 for 8 days	3 × 10 repeitions @ 115%1RM eccentric back squats Performed on d5 supplementation	= Muscle soreness ↓ Blood markers of muscle damage (CK) ↓ Blood marker inflammation (IL6)
Ives et al. [136]	Recreationally active men RCT	Carbohydrate (*n* = 14), whey protein (31 g, *n* = 16), whey protein (31 g) and berry powder (100 mg, *n* = 17), consumed immediately, 6 and 22 h post-ex (low PP diet consumed day before and day of exercise)	100 maximal eccentric knee extensor contractions	↑ Knee extensor strength ↓ Muscle soreness No significant additive effects of berry extract except for lower soreness.
McLeay et al. [117]	Recreationally active women (*n* = 10) Crossover trial	200 g blueberry in test beverage (~ 420 mg PP, 242 mg anthocyanins) consumed × 3 on day of exercise and × 1 am d1 and d2 post-ex	3 × 100 unilateral maximum knee extensor eccentric contractions Performed 10 h after first dose	↑ Knee extensor strength = Muscle soreness = Blood markers of muscle damage (CK) = Blood markers of inflammation (IL6) = Blood markers of oxidative damage (PC, p = 0.06)
Peschek et al. [118]	Endurance trained (*n* = 8) Crossover trial	Chocolate milk (1 g·kg^−1^ CHO, 0.3 g·kg^−1^ protein, 350 mg cocoa flavanols) versus chocolate milk with no cocoa flavanols; consumed 1 and 2 h post-ex	30 min downhill running	= Knee extensor muscle strength and 5 km time trial = Muscle soreness = Blood markers of muscle damage (CK)
Romain et al. [148]	Recreationally active (*n* = 13) Crossover trial	1.5 g.d^−1^ extract from mangosteen, black elderberry and pomegranate (219 mg PP, 130 mg ellagic acid and derivatives, 85 × anthone derivatives) × 1 for 5 days (first dose on day of exercise)	8 × 8RM half squats	↓ Muscle soreness ↓ Blood markers of muscle damage (Mb)

↑ Increased, ↓ decreased, = no change, *CHO* carbohydrate, *CRP* C reactive protein, *CK* creatine kinase, *IL6* interleukin 6, *ex* exercise, *incl* including, *LDH* lactate dehydrogenase, *Mb* myoglobin, *PC* protein carbonyls, *PP* polyphenol, *RCT* randomised controlled trial with parallel groups, *RM* repetition maximum, *TBARS* thiobarbituric acid reactive substances

The effect of Montmorency cherry supplementation on muscle soreness after intensive exercise has been assessed in eight studies, and soreness measured using a visual analogue pain scale [104, 107, 112] or pressure pain tolerance [109] was reduced in half of these studies. There is no clear pattern to explain this variation across studies: in two studies favourable effects on both recovery of muscle strength and soreness were evident [104, 107]; in others favourable effects on muscle function but not soreness were observed [105, 106]; and Beals et al. [110] and McCormick et al. [111] found no effects on either soreness or muscle function, but the exercise protocol did not induce loss of muscle strength or soreness. Lastly, Kuehl et al. [112] found significant reductions in muscle soreness in runners who consumed Montmorency cherry juice (1200 mg polyphenols) for 7 days prior to and on the day of the Hood to Coast relay race, but changes in muscle function were not assessed. Suppression of inflammation induced by muscle damage is the proposed mechanism by which polyphenol supplementation may attenuate muscle soreness [113]. At present measures of serum markers of inflammation are available for some but not all studies, and there seems to be no relationship between reduced soreness after Montmorency cherry supplementation and serum markers of inflammation. Levers et al. [109] found reduced soreness but no effect on serum TNFα, IL1β, IL6 or IL8; Kuehl et al. [112] and Connolly et al. [104] found reductions in soreness but no serum inflammatory marker data are available. Howatson et al. [105] found reduced serum IL6 and C-reactive protein (CRP) after cherry supplementation but no significant effect on soreness; only Bell et al. [107] found reductions in both soreness and serum IL6 response, but serum TNFα, IL1β, IL8 and CRP were not affected by Montmorency cherry supplementation. Several studies found no effects of Montmorency cherry supplementation on serum markers of inflammation [106, 110, 111], but these involved unilateral and/or relatively small muscle group exercise, which did not result in elevations in systemic markers of inflammation. Therefore, the absence of polyphenol effects in these studies is unsurprising. By definition, soreness is a highly subjective measure even when pressure pain tolerance is measured using an algometer, hence although important for athlete performance it is difficult to reliably and objectively quantify. The quantification of inflammation within the damaged muscle itself, alongside measures of muscle soreness, could be an important step forward in understanding the effects of polyphenols.

Pomegranate juice consumption has also been shown to enhance recovery of elbow flexor [114–116], and knee extensor [116] muscle function after intensive exercise in recreationally active men. However, Trombold et al. [115] found that in resistance-trained men, pomegranate juice (250 ml providing ~ 620 mg polyphenols) consumption twice per day for 7 days prior to and after exercise resulted in faster recovery of elbow flexor but not knee extensor isometric strength compared to placebo, after muscle damage was induced by eccentric elbow flexor and knee extensor exercise. The knee extensors were relatively refractory to muscle damage (15–20% reduction in isometric strength vs. 25–35% loss of elbow flexor isometric strength) in this population, which may contribute to the lack of polyphenol effects in knee extensors in this population. In contrast, Machin et al. [116] found that recovery of both elbow flexor and knee extensor strength in recreationally active men was enhanced by consumption of pomegranate concentrate providing 650 mg polyphenols either once or twice per day for 3 days prior to exercise, suggesting that the lower polyphenol dose was equally effective.

Only Trombold et al. [115] found that pomegranate supplementation reduced muscle soreness, specifically after eccentric elbow flexor exercise in resistance-trained men, but no serum markers of inflammation were measured in this study. Trombold et al. [114] and Machin et al. [116] measured muscle damage markers myoglobin (Mb) and/or creatine kinase (CK) and found that the exercise-induced increases were not affected by pomegranate supplementation. Serum markers of inflammation were measured in only one study [114], and there were no effects of pomegranate supplementation. However, as observed for Montmorency cherry supplementation studies, damage to a relatively small muscle group (elbow flexors) was not sufficient to induce any change in serum IL6 or CRP, hence it is unsurprising that polyphenol effects were not detectable.

Blueberry supplementation consumed in the form of a smoothie on the day of exercise (1360 mg polyphenols) and during 2 days of recovery (420 mg polyphenols per day) enhanced recovery of knee extensor strength after unilateral eccentric exercise in recreationally active women, but did not reduce muscle soreness or serum IL6 although there was a strong tendency for reduced oxidative modification of serum proteins (protein carbonyls) [117]. Conversely, Peschek et al. [118] found no effect of the addition of cocoa flavanols to chocolate milk in the recovery of muscle strength or 5-km time trial performance or muscle soreness in eight endurance-trained males, when consumed 1 and 2 h after downhill running. However, this finding in this randomised crossover trial is confounded by the use of downhill running to induce muscle damage, with all second trials likely to be affected by repeated bout effect, irrespective of treatment [119]. With the exception of McCormick et al. [111], all other studies reported here either adopted unilateral exercise for crossover trials to minimise repeated bout effects or utilised parallel group design to avoid repeated bout effects, although such studies are susceptible to the high degree of inter-individual variation evident in muscle damage and soreness responses.

### Mechanisms of Action

Serum markers of oxidative damage (TBARS, MDA, lipid hydroperoxides, F2-isoprostanes, protein carbonyls, nitrotyrosine) were measured in five of the nine studies that found favourable effects of polyphenol supplementation on recovery of muscle function. Oxidative damage was suppressed either significantly (TBARS [105]; protein carbonyls [106]) or there was a strong non-statistically significant trend (lipid hydroperoxides [107]; protein carbonyls [117]) or in only one study there was no effect (lipid hydroperoxides [108]). A number of other studies have found reductions in oxidative damage markers after intensive exercise with prior polyphenol supplementation, although changes in muscle function were not measured (Montmorency cherry, 1300 mg·day^−1^ polyphenols [120]; blackcurrant, 240 mg anthocyanin [87]; pomegranate juice, 2560 mg·day^−1^ polyphenols [121]). As described earlier (Sect. 2.1), it seems unlikely that polyphenols serve as direct antioxidants due to their relatively low concentration relative to endogenous antioxidants in serum and tissue. It is more likely that chronic supplementation (> 3 days) reduces serum markers of oxidative damage via upregulation of endogenous antioxidants through Nrf2 and antioxidant response element pathway signalling [24, 122]. Tart cherry juice consumption has been shown to increase hepatic superoxide dismutase and glutathione peroxidase activity in mice [123]. Supplementation with anthocyanins isolated from purple sweet potato increased Nrf2 gene expression and Nrf2 nuclear translocation in rat liver [124]. Charles et al. [125] recently demonstrated that the age-related increases in oxidative stress and declines in mitochondrial function in mice were prevented by supplementation with an anthocyanin-rich extract derived from red grapes. This was achieved both via reduced reactive oxygen species generation as well as increased endogenous antioxidant enzyme gene expression. Dark chocolate supplementation for 3 months has also been shown to enhance superoxide dismutase and catalase expression in muscle of patients with heart failure and type 2 diabetes [48], and more recently has been shown to reduce carbonylation of proteins within skeletal muscle of healthy adults [126]. In contrast, McLeay et al. [117] only commenced blueberry supplementation 10 h prior to intensive exercise and hence increased endogenous antioxidant capacity is an unlikely explanation of the enhanced restoration of muscle function and a strong trend for lower serum protein carbonylation. Blueberry supplementation has been shown to suppress neutrophil NADPH oxidase activity [14], one of the key sources of superoxide production, which may contribute to the observed effects. A range of polyphenols have been shown to inhibit superoxide producing enzymes, such as NADPH oxidase and xanthine oxidase (for review, see Maraldi [47]).

Polyphenols have been shown to inhibit cyclo-oxygenase activity (COX1 and COX2) in similar fashion to non-steroidal anti-inflammatory drugs, and there is extensive in vitro and in vivo evidence of anti-inflammatory effects of polyphenols (for review see Peluso et al. [127]). The important and complex contribution of inflammatory pathways to the healing and remodelling process suggests the anti-inflammatory effects of polyphenols may be a key component of the mechanisms of action. Ten of the reviewed studies included serum markers of inflammation, with polyphenol-induced reductions in serum IL6 response to intensive exercise in five studies [34, 105, 107, 108, 120]). However, muscle soreness was attenuated in only one of these studies [107], whilst recovery of muscle function was enhanced in both studies where isometric force and inflammatory marker data were available [105, 107]. In four studies [106, 110, 111, 114], there was no effect of polyphenol supplementation on serum markers of inflammation; however, the exercise protocol employed did not induce elevation in any serum markers in these studies. In the remaining studies, polyphenol supplementation did not affect serum markers of inflammation despite enhanced recovery of muscle function [117] and reduced soreness [109]. The reliance on proxy serum markers of inflammation does not provide sufficient sensitivity to understand processes taking place within the damaged muscle itself. This point was clearly confirmed by Jajtner [128], since supplementation with a proprietary blend of green and black tea polyphenols suppressed IL8 protein expression in human skeletal muscle after intensive exercise, but there was no effect on circulating IL8 concentration. Myburgh et al. [129] investigated the effect of grape seed-derived proanthocyanidolic oligomer (PCO) supplementation on the healing process after hindlimb contusion injury in male Wistar rats. PCO treated rats exhibited reduced neutrophil activation on day 1, normal magnitude but earlier macrophage response and earlier satellite cell activation. However, equivalent data from studies with human participants are not yet available.

Sleep is an important component of an athlete’s programme to ensure optimal recovery. However, use of stimulant supplements, evening training sessions and matches, travel and other aspects of an athlete’s busy life can compromise both sleep quantity and quality. There is some evidence to suggest that Montmorency cherry [130, 131] and Jerte valley [132, 133] cherry supplementation improves sleep quality (assessed via accelerometry and sleep questionnaire), which may also contribute to the observed benefits for recovery. Modulation of the pro-inflammatory cytokine response to exercise has been suggested as one possible mechanism by which sleep quality may be improved, as well as the melatonin content of cherries. Elevation of urinary melatonin metabolites after 7 days’ cherry supplementation in parallel with enhanced sleep quality supports this assertion. However, in these studies supplements were consumed in the morning and evening whereas melatonin supplements are normally prescribed to be consumed prior to sleep, and in addition the dose provided by cherries is relatively low (85 µg vs. 2–3 mg clinical dose) and there is some scepticism surrounding this proposition [134]. Nevertheless, recent data from in vivo rat studies suggest that co-ingestion of melatonin with caffeic acid and/or quercetin enhances melatonin pharmacokinetics [135]. More human in vivo studies are needed to verify the somniferous effects of Montmorency cherry and other polyphenols and improve understanding of the mechanism.

## Methodological Limitations and Future Work

The existing literature is flawed by a number of limitations that preclude the identification of optimal polyphenol dosing strategies for desired outcomes and also constrain understanding of the mechanisms of action. A key challenge in utilising natural fruit-derived supplements is that the polyphenol blend is influenced by the plant species, growing conditions and post-harvest processing. As a consequence, the polyphenol content of supplements will vary from batch to batch, but relatively few of the published studies provide detailed composition of the batch specific polyphenol blend consumed. This is essential for future work to ensure the accuracy of dose response data and hence our ability to identify optimal polyphenol doses and blends.

Dietary controls are a further factor worthy of consideration. Several of the reviewed studies incorporated some element of dietary polyphenol restriction [11, 64, 67, 80, 82, 107, 120, 136], in attempts to reduce the background noise that may be introduced by variation in dietary polyphenol intake, but which may also maximise the effects produced by polyphenol supplementation. Habitual polyphenol intake is difficult to accurately quantify, since despite our knowledge that polyphenol content of foods will be highly variable, reliance on standard values is required (http://phenol-explorer.eu/ is an excellent resource for this purpose). Accuracy is also influenced by the known proclivity for under- and over-reporting of food intake using diet diaries. Population estimates of average intakes are diverse depending upon the plant content of diets, with total polyphenols ranging from 380 to 1400 mg [137], anthocyanins ~ 60 mg·day^−1^, flavonols 20–35 mg·day^−1^, catechins 18–50 mg·day^−1^, hydroxycinnamic acids 800 mg·day^−1^ (coffee drinkers) (for a review, see Manach et al. [138]) and proanthocyanidins ~ 95 mg·day^−1^ [139]. Many of the studies reviewed here provide > 1 g of polyphenols per day containing high levels of anthocyanins (cherry, blueberry, blackcurrant 200–550 mg) or ellagitannins (pomegranate 600 mg), which is many-fold higher than habitual intake. For this reason and to ensure the ecological validity of the data, it would seem that superimposing polyphenol supplementation onto the backdrop of habitual diet is the most appropriate approach.

Polyphenol chemistry is highly complex, but to date, studies in which the recovery and performance effects of polyphenol supplementation have been assessed have not included quantification of the plasma phenolics. Such measurements may allow identification of the bioactive metabolites and inform optimisation of the polyphenol blends consumed.

Our understanding of the mechanisms by which polyphenols exert favourable effects on performance and recovery is limited by the dearth of data on processes occurring within muscle. Published studies have relied upon proxy markers within serum to inform understanding of the antioxidant and anti-inflammatory processes that seem likely to underpin the beneficial effects observed. As described recently by Close et al. [140], many of the serum markers of oxidative damage are flawed and over-simplify the complexity of redox regulation. Certainly, blood markers are a poor surrogate for direct analysis of muscle tissue. To further progress understanding, studies incorporating muscle biopsies are needed that allow direct assessment of signalling via Nrf2 and ARE pathways and changes in antioxidant enzyme activity in muscle after polyphenol supplementation.

The controversy with regard to antioxidant vitamin supplementation and training adaptation continues, with evidence of no effect [141] or attenuation of adaptation [142]. It is clear that ROS and inflammatory pathways are implicated in the cell-signalling pathways that drive training adaptation, so it is plausible that if these signals are suppressed by antioxidant supplementation, ergolytic effects will result. However, in contrast to antioxidant vitamins and minerals, the antioxidant effects of polyphenols do not seem to arise from radical scavenging but rather from up-regulation of endogenous antioxidant systems. Different effects might therefore be anticipated between vitamins and polyphenols; however, there is a lack of empirical data. Taub et al. [126] recently confirmed previous exercise mimetic observations in mice [143], by which supplementation with 20 g of epicatechin-rich cocoa for 3 months resulted in increased ${\dot{\text V}}{\text O}_{2}\max$ and significant increases in skeletal muscle 5’ adenosine monophosphate-activated protein kinase (AMPK), PGC1α and citrate synthase protein levels. A small number of training studies with human participants have been performed with isolated resveratrol supplementation with largely detrimental consequences either for performance [144–147] or cellular level [145, 147] adaptation. However, no studies have yet been published in which the effects of a fruit-derived blend of polyphenols on training adaptation have been investigated.

## Practical Application

From a practical perspective, this review provides data to allow sport nutrition and sport science practitioners to make recommendations to coaches and athletes regarding the efficacy of polyphenols to improve performance and aid recovery from their chosen sport or training discipline. The results from several studies suggest that acute and chronic polyphenol supplementation is associated with an improvement of performance with no reported adverse side effects. Additionally, it appears that supplementation with fruit-derived polyphenols will assist in the recovery of muscle function and reduce muscle soreness following intensive exercise.

In particular, athletes participating in sports that involve time trials (cycling) and repeated sprints (field and court sports) may experience performance gains when ingesting an acute dose of polyphenols (1–2 h prior to competition) or supplementing polyphenols chronically (1–6 weeks prior to competition). Similarly, athletes participating in training sessions that involve the completion of repeated sprints (6–30 s) and repeated high-intensity interval training bouts may consider supplementing with polyphenols as improvements during training may translate to sporting performance although this remains to be experimentally demonstrated. Since performance effects were of similar magnitude for acute and chronic supplementation, current evidence would suggest that acute supplementation with ~ 300 mg polyphenols 1–2 h prior to exercise may be ergogenic. Whilst research supports the use of polyphenols in conjunction with high-intensity training, there is currently a lack of evidence to support its use in conjunction with resistance training.

As athletes’ training and competition schedules increase, the optimisation of recovery post-exercise is imperative. The compounding effects of successive training sessions and/or competitive bouts on physiological and physical function may be attenuated with the use of polyphenols. Current evidence would suggest that supplementation with > 1000 mg polyphenols per day for 3 or more days prior to and following exercise will enhance recovery following sporting events that induce muscle damage, such as heavy resistance training, and for tournament sports that involve successive matches or rounds with short recovery periods. Consumption of 450 g blueberries, 120 g blackcurrants or 300 g Montmorency cherries would approximately provide this dose [3]. Practitioners should consider their athletes’ personal taste preferences and lifestyle demands when recommending individual supplementation protocols to ensure compliance.

## Conclusions

In summary, there is growing evidence that acute and chronic supplementation with fruit-derived polyphenols may enhance exercise performance, with the mechanisms most likely to be related to antioxidant and vascular effects. However, this research is at an early stage and more work is required to optimise dosing strategies and to determine the specific modes, intensities and durations of exercise for which ergogenic effects may be achieved. There is a larger body of evidence that suggests that chronic polyphenol consumption enhances recovery from intensive exercise. More research is still required to identify the optimal dose and blend of polyphenols to support recovery, and ideally future studies will measure processes within muscle as well as plasma phenolic concentrations so that the specific bioactive compounds and the mechanisms of action can be identified.
